# Subsidence‐Derived Volumetric Strain Models for Mapping Extensional Fissures and Constraining Rock Mechanical Properties in the San Joaquin Valley, California

**DOI:** 10.1029/2020JB019980

**Published:** 2020-09-11

**Authors:** Grace Carlson, Manoochehr Shirzaei, Chandrakanta Ojha, Susanna Werth

**Affiliations:** ^1^ School of Earth and Space Exploration Arizona State University Tempe AZ USA; ^2^ Department of Geosciences Virginia Tech Blacksburg VA USA; ^3^ Positioning and Community Safety Division Geoscience Australia Canberra ACT Australia; ^4^ School of Geographical Sciences and Urban Planning Arizona State University Tempe AZ USA

**Keywords:** San Joaquin Valley, drought, groundwater, volume strain, hazard

## Abstract

Large‐scale subsidence due to aquifer‐overdraft is an ongoing hazard in the San Joaquin Valley. Subsidence continues to cause damage to infrastructure and increases the risk of extensional fissures.Here, we use InSAR‐derived vertical land motion (VLM) to model the volumetric strain rate due to groundwater storage change during the 2007–2010 drought in the San Joaquin Valley, Central Valley, California. We then use this volumetric strain rate model to calculate surface tensile stress in order to predict regions that are at the highest risk for hazardous tensile surface fissures. We find a maximum volumetric strain rate of −232 microstrain/yr at a depth of 0 to 200 m in Tulare and Kings County, California. The highest risk of tensile fissure development occurs at the periphery of the largest subsiding zones, particularly in Tulare County and Merced County. Finally, we assume that subsidence is likely due to aquifer pressure change, which is calculated using groundwater level changes observed at 300 wells during this drought. We combine pressure data from selected wells with our volumetric strain maps to estimate the quasi‐static bulk modulus, *K*, a poroelastic parameter applicable when pressure change within the aquifer is inducing volumetric strain. This parameter is reflective of a slow deformation process and is one to two orders of magnitude lower than typical values for the bulk modulus found using seismic velocity data. The results of this study highlight the importance of large‐scale, high‐resolution VLM measurements in evaluating aquifer system dynamics, hazards associated with overdraft, and in estimating important poroelastic parameters.

## Key Points

- Models of volume strain rate are developed for the San Joaquin Valley during the 2007–2010 drought
- High risk of fissure initiation occurs on the periphery of subsiding zones based on the ratio of tensile stress to soil tensile strength
- The quasi‐static bulk modulus is estimated to be 0.19 GPa

## 1 Introduction

Groundwater is an essential resource for agriculturally productive regions across the world that suffer from low annual precipitation and limited surface water availability. The Central Valley in California holds the second largest underground aquifer in the United States, which delivers an essential proportion of the freshwater supply in central California. The presence of this aquifer has supported extensive irrigation of cropland since the late 1800s, allowing it to become one of the most agriculturally productive regions in the United States (Faunt, 2009). Recent increases in groundwater withdrawal are linked to replacing seasonal crops with perennial crops and orchards, which are more profitable, but also more water‐intensive (Borchers et al., 2014). Unfortunately, this historic and modern groundwater use has caused a steady decline of water tables in the Central Valley, particularly in the dryer San Joaquin Valley, located in the southern half of the Central Valley. Lowering of water tables has forced deeper well drilling, which is costly and more energy‐intensive (Perrone & Jasechko, 2019).

In response to dropping groundwater levels, a large portion of the San Joaquin Valley also experiences land subsidence (Farr et al., 2015; Ojha et al., 2018; Poland et al., 1975). Subsidence due to the removal of fluid is widespread and well documented, occurring in many locations worldwide, for example, in southern Texas (Buckley et al., 2003), Las Vegas, Nevada (Holzer & Pampeyan, 1981), central Arizona (Holzer et al., 1979; Holzer & Pampeyan, 1981; Miller et al., 2017; Miller & Shirzaei, 2015) in the United States; as well as in the Mekong River Delta in Vietnam (Erban et al., 2014); and in Central Mexico (Chaussard et al., 2014). Although subsidence can be the result of many different processes including hydrocarbon extraction (Holzer & Bluntzer, 1984), geothermal power production (Mossop & Segall, 1999), permafrost melting (Nelson et al., 2001), and tectonics (e.g., Moxon & Graham, 1987), in the San Joaquin Valley, it is largely due to groundwater extraction.

Land subsidence began in the San Joaquin Valley in the early 20th century, and by 1975, approximately half of the Valley was experiencing subsidence (Poland et al., 1975). In some locations, the land surface dropped more than 850 cm between 1926 and the 1970s (Poland et al., 1975). A recent study showed subsidence of more than 2.5 cm over an area of 3,100 km^2^ in the San Joaquin Valley with maximum subsidence of over 50 cm near the town of El Nido from January 2008 to January 2010 (Sneed et al., 2018). Ojha et al. (2018) showed subsidence rates of ~25 cm/yr during the 2007–2010 drought in the vicinity of the Tulare Lake region. This subsidence continues to cause expensive infrastructure damage including reduced flow capacity in canals, damage to irrigation pipelines, reduced effectiveness of levees, and damage to roads, bridges, and building foundations (Borchers et al., 2014; Hernandez‐Marin & Burbey, 2010; Holzer & Bluntzer, 1984; Sneed et al., 2018). Damage to canals, bridges, irrigation pipelines, and other structures in the San Joaquin Valley has cost more than 1.3 billion dollars (2013 dollars) in repairs (Borchers et al., 2014).

Continued subsidence also has the potential to cause hazardous extensional fissures to develop at or near the surface (Hernandez‐Marin & Burbey, 2010; Holzer & Bluntzer, 1984). Fissure initiation is often attributed to lateral variability in aquifer thickness, differential pumping and spatially variable subsidence patterns, or bedrock topography and preexisting faults that create groundwater flow barriers (Jachens & Holzer, 1982; Sheng & Helm, 1998). Fissures have the potential to damage subsurface structures and cause contamination of groundwater as they may direct potentially contaminated overland flow into subsurface channels. Although documentation of fissures in the San Joaquin Valley is scarce, three known fissures were discovered at the surface near Pixley, California, in 1969. At least one of these fissures was attributed to differential compaction caused by groundwater extraction (Holzer & Bluntzer, 1984). A recent study using geophysical logs from the California Department of Conservation, Division of Oil, Gas, and Geothermal Resources found possible subsurface vertical offsets in the vicinity of the Pixley fissure (Rucker et al., 2019). The authors speculate that this compaction fault may have created a hydraulic barrier, causing differential subsidence and strain accumulation on one side of the fault, which lead to the fissure's formation (Rucker et al., 2019). Fissures are common in other places with significant groundwater extraction‐induced subsidence in the southwestern United States, such as Phoenix, Arizona, and Las Vegas, Nevada (Borchers et al., 2014; Holzer, 1981). Once a fissure is initiated, extension of the fissure head and erosion of the fissure walls can cause additional damage, making them long‐lived hazards.

Here, we evaluate hazards associated with subsidence and extensional fissuring during the recent 2007–2010 drought. Estimates of total groundwater loss during this drought range from 20.4 to 31.0 km^3^ (Famiglietti et al., 2011; Ojha et al., 2018; Scanlon et al., 2012). We use vertical land motion (VLM) measurements obtained from a combination of Interferometric Synthetic Aperture Radar (InSAR) and Global Positioning System (GPS) data (Ojha et al., 2018) (section 2.1) to model the volumetric strain rate as a result of compaction of the San Joaquin Valley aquifer system due to groundwater pumping (sections 3 and 4.1). Next, we recognize that the spatial variability in subsidence rates can promote tensile fissures, and we use our volumetric strain rate model to create a map showing areas of the Valley with the most significant hazard (section 4.2). Finally, we combine our volumetric strain rate model with estimates of groundwater pressure change obtained from wells (section 2.2) to calculate the quasi‐static bulk modulus (section 4.3) useful for low‐frequency deformation, such as aquifer compaction, that is a result of pressure change due to pumping. We compare our estimates with a bulk modulus derived from seismic velocity data (described in section 2.3).

### 1.1 Geologic and Hydrologic Setting

California's Central Valley is a large alluvial basin nestled between the Coast Ranges to the west and the Sierra Nevada Mountains to the east. Basin‐fill deposits thicken to the west, where they can be more than 15 km deep (Faunt, 2009). They are composed of sand and gravel‐sized sediment interbedded with clay and silt deposits that can make up to half of the overall thickness in some locations (Faunt, 2009; Planert & Williams, 1995). The northern Sacramento Valley and southern San Joaquin Valley are split by the San Francisco Bay/Sacramento‐San Joaquin River Delta, the confluence of the Sacramento River flowing from the north and the San Joaquin River flowing from the southeast (Faunt, 2009). Towards the southern end of the San Joaquin Valley lies the internally drained Tulare Basin, which is fed by several rivers sourced from the Sierra Nevada (Faunt, 2009). Here, we focus on the San Joaquin Valley, where historically and presently, we find the largest subsidence.

The San Joaquin Valley aquifer system is composed of an upper semi‐confined unit and a lower confined unit on the western side of the Valley, with the Corcoran Clay forming the confining unit between them (Faunt, 2009). The upper semi‐confined unit is up to ~150–250 m thick and contains alluvial deposits from rivers draining from the Coast Ranges and the Sierra Nevada Mountains ranging in size from gravel to sand. Fine‐grained lenses can be found throughout the Valley (Faunt, 2009). The lower confined aquifer contains similar coarse‐grained sand and gravels, particularly in approximately the southwestern quadrant. Sediments fine somewhat towards the Valley axis, where on average coarse‐grained deposits only make up ~10–40% of the total thickness (Faunt, 2009). The ratio of coarse to fine sediments in this lower aquifer also increases to the south (Faunt, 2009). The Corcoran Clay is a Pleistocene‐aged laterally continuous lacustrine deposit that can be up to 70 m thick, found at depths between ~30 and 275 m. It is thickest on the west and south‐southwest portions of the San Joaquin Valley (Page, 1977). This layer is of extremely low permeability, naturally preventing vertical flow between the upper and lower aquifers. The fine‐grained lenses and Corcoran Clay confining unit are highly susceptible to compaction (Faunt, 2009; Williamson et al., 1989).

The San Joaquin Valley maintains a semi‐arid to arid climate, with precipitation averaging ~10–30 cm per water year (PRISM Climate Group, 2019; 30‐yr normals) and higher precipitation during the wet season (December–March) than the dry season (June–September) (PRISM Climate Group, 2019; 30‐yr normals). Groundwater pumping accounts for ~30–40% of water use. In times of drought, when surface water availability is lower, it can account for nearly 70% (Borchers et al., 2014; Faunt, 2009). Pumping has also altered groundwater flow directions to be primarily vertical, aided by perforated well casings hydraulically connecting the confined and semi‐confined units through the fine‐grained lower conductivity units (Faunt, 2009). Annual recharge and discharge of the aquifer is largely controlled by irrigation activity in much of the San Joaquin Valley (Faunt, 2009). However, some recharge also occurs through losing streams coming from the Sierra Nevada Mountains.

### 1.2 Linking Groundwater Withdrawal to Subsidence

The relationship between groundwater pumping and subsidence can be explained using the aquitard drainage and aquifer compaction model (Galloway & Burbey, 2011), based on the principle of effective stress (Terzaghi, 1925) and hydrodynamic consolidation theory (Holzer, 1981). The principle of effective stress explains that the total stress (σ) on a saturated, porous, matrix is partially relieved by fluid pressure (*P*) in the pore space:

(1) σ′=σ−P

where *σ*′ is the effective stress felt on the matrix. Assuming minimal tectonic stress over short time scales, the total stress is simply the vertical principal stress, or weight of the overburden. As groundwater is pumped, the fluid pressure is reduced, thus increasing the stress felt on the grains in the matrix. If we assume that the overburden load is constant, then the change in effective stress is simply the opposite of the change in pore pressure, which is measurable through groundwater level observations (Holzer, 1981).

Hydrodynamic consolidation theory explains that differences in mechanical properties of the sediment, such as compressibility and permeability, determine the different deformation responses of coarse‐grained aquifer units and fine‐grained aquitard layers to increases in effective stress. As water is pumped from coarser grained aquifer units, there is pressure change in the aquifer system. If pressure change in the aquifer system is large enough, fine‐grained sediments may rearrange and collapse pore space. If the change in pore pressure exceeds the previous maximum vertical effective stress, known as pre‐consolidation stress, then these pore spaces may collapse permanently, resulting in inelastic deformation. Additionally, because hydraulic diffusivity in the fine‐grained layers is low, a residual drainage and delayed compaction response may occur in the aquitard and cause a delayed subsidence at the surface. In contrast, coarse‐grained aquifer units are less susceptible to compaction and behave elastically under most pressure changing conditions. Thus, aquifer systems with thick fine‐grained lenses are more susceptible to permanent compaction and storage loss, which is reflected as permanent subsidence at the surface. As long as pre‐consolidation stress is not exceeded, though, the deformation is recoverable (Galloway & Burbey, 2011). It has been shown that pumping in the San Joaquin Valley during recent droughts has caused inelastic deformation, permanently reducing aquifer storage (Chaussard & Farr, 2019; Ojha et al., 2019; 2020).

## 2 Data

### 2.1 Vertical Land Motion

**Figure 1:**
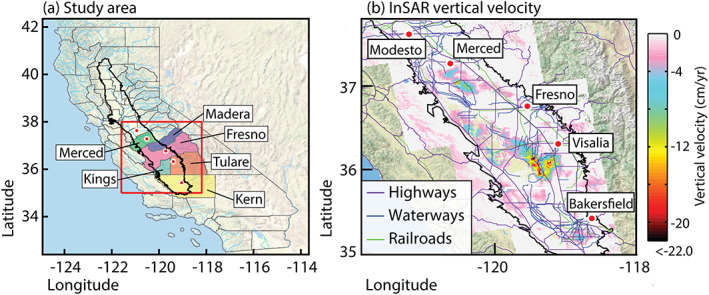
Study region and vertical velocity. (a) Area of interest denoted by the red square. California counties outlined in gray and counties that fall within the study region are identified. The Central Valley is outlined in black. Red circles show locations of cities in the San Joaquin Valley and labeled in (b). (b) InSAR‐derived vertical velocity using ALOS‐1 SAR images collected from 24 December 2006 to 1 January 2010 after Ojha et al. (2018). San Joaquin basin outlined in black overlain with highways in purple, canals and aqueducts (waterways) in blue, and railroads in green. Cities are shown as red circles and labeled.

VLM estimates are from Ojha et al. (2018). To achieve VLM across the entire Central Valley, they combined 420 SAR images from ascending tracks of the ALOS‐1 L‐band satellite collected from 24 December 2006 to 1 January 2010 with horizontal displacements from GPS stations of the Plate Boundary Observatory (PBO) network collected over the same time period. The authors generated InSAR LOS displacements using a multi‐temporal Wavelet‐Based InSAR (WabInSAR) algorithm (Shirzaei, 2013; Shirzaei & Bürgmann, 2012). To this end, several wavelet‐based analyses were applied to the interferograms to identify and remove noisy pixels and reduce the effects of topographically‐ correlated atmospheric phase delay (Shirzaei, 2013) and spatially uncorrelated DEM error (Shirzaei, 2013; Shirzaei & Bürgmann, 2012). A reweighted least‐squares approach was used to invert for LOS velocities from more than 1,600 stacked interferograms. The LOS velocities were then combined with horizontal GPS velocities to achieve VLM velocity. Non‐tectonic horizontal velocities in the San Joaquin Valley are minimal compared to vertical, as mentioned in previous work (e.g., Farr et al., 2015; Ojha et al., 2018); therefore, only the vertical is considered here. Additional details on interferogram generation, achieving LOS velocity, and combining LOS velocity with horizontal GPS velocities to achieve VLM velocity can be found in Ojha et al. (2018). The authors also validate their InSAR‐derived VLM velocity against GPS vertical velocity and find an average standard deviation of ~0.8 cm/yr for their differences across the entire Central Valley. Here, we are interested only in the San Joaquin Valley, so we remove pixels north of 38° latitude. In addition, we are using only the vertical velocity, although we recognize that the deformation changes throughout the time period of interest. For example, in the summer of 2008, the subsidence rate increased dramatically, particularly near Corcoran (Farr et al., 2015). This continued until it slowed in the winter of 2009/2010 (Farr et al., 2015). Small seasonal fluctuations in both groundwater levels and VLM also occur during the drought with an equivalent water height ranging from ~20 to 100 cm and an average VLM amplitude of ~1.5 cm, peaking in late winter to early spring (Carlson et al., 2020; Ojha et al., 2018). The resulting VLM velocity is shown in Figure 1.

### 2.2 Groundwater Observation Wells

**Figure 2:**
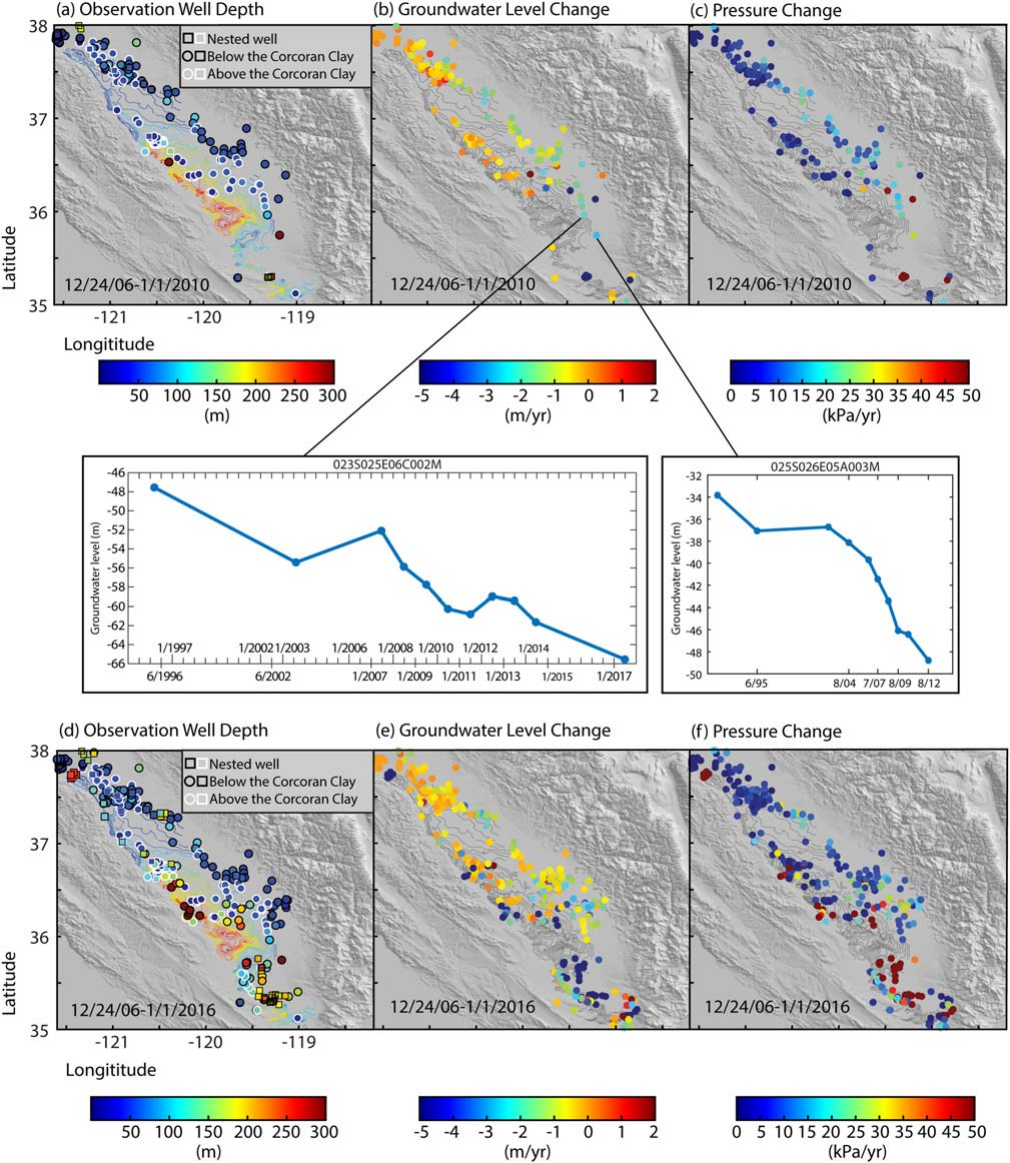
Groundwater level data from the 2007–2010 drought (a–c) and from +6 years from the end of the drought (d–f). (a, d) Groundwater observation well depth mapped on contours of depth to the Corcoran Clay shown in the same color scale obtained from the U.S. Geological Survey. Observation wells with white rings show those with depths above the Corcoran Clay layer while black rings show observation wells below the clay layer. Squares show nested wells. (b, e) Groundwater level change rate in meters. The locations of the two well observation timeseries are shown in (b). These show groundwater level with respect to the ground surface. (c, f) Absolute pressure change rate. Shaded contours are of the Corcoran Clay, same as (a, d). All groundwater observation data are obtained from the California Department of Water Resources (https://data.ca.gov/dataset/) and the U.S. Geological Survey (https://waterdata.usgs.gov/nwis/gw).

We collected 1,098 groundwater level time series that were recorded at observation wells from the California Department of Water Resources (2019) and the U.S. Geological Survey (2019). Of those time series, only 300 contain data during our observation period (24 December 2006 to 1 January 2010). Of those 300 time series, 18 have no well depth listed, and 171 are located either outside of the Corcoran Clay extent or below the Clay layer (111 are above the Clay layer). After the end of the drought (1 January 2010 to 1 January 2016), 508 more timeseries were added, 35 of which have no well depth listed and 374 are below or outside of the Corcoran Clay. Figure 2 shows the distribution of groundwater level observations in the San Joaquin Valley. Wells to the north and west generally show a lower rate of decline but are also some of the shallowest wells and therefore are likely not observing the largest groundwater level changes. Two examples of groundwater level observation (measured w.r.t ground surface elevation) timeseries are shown in Figure 2b. These timeseries are of wells below or outside of the Corcoran Clay and near the area of greatest subsidence (Figure 1b). We can see rapid drawdown during the drought period in both wells, as well as an increase in the frequency of observations. This irregular temporal sampling is typical for many wells in the San Joaquin Valley.

Although groundwater level observations below the Corcoran Clay are inconsistent both spatially and temporally, we can still extract valuable information about pressure changes within the aquifer. Using the change in groundwater level (Δ*h*), one can estimate the change in pressure (Δ*P*) using the equation (Holzer, 1981).

(2) $$\Delta P=\rho_{w}g\Delta h$$

where *ρ*_*w*_ is the density of water and *g* is the gravitational acceleration. Well depth, groundwater level change, and absolute pressure change rate are shown in Figure 2 for the 2007–2010 drought (a–c) and 2007–2015 (d–f). The 2007–2015 period also contains the drought period from 2012–2015, which was even more severe than the 2007–2010 drought. Comparing these two time periods, we can see that many deeper observation wells begin collecting data after the 2007–2010 drought. This is partially a result of passage of the Sustainable Groundwater Management Act in 2014, which requires groundwater basins in California to maintain sufficient groundwater monitoring networks (CDWR, 2016).

### 2.3 Seismic Velocity

Seismic velocities are commonly used to solve for the bulk modulus, a mechanical parameter that is the ratio of applied stress to resulting volumetric strain (Mavko et al., 2009). Here, we use seismic velocity data to calculate the dynamic bulk modulus in order to compare to our volume‐strain derived bulk modulus, also known as the quasi‐static bulk modulus. The difference between these two moduli is the rate of deformation at which they are estimated; while the dynamic bulk moduli corresponds to a condition under which deformation occurs at a high rate, the quasi‐static bulk modulus corresponds to a much slower deformation process. In order to solve for the dynamic bulk modulus, we use *P* wave seismic velocity data obtained from Lin et al. (2010). They used first‐arrival and differential times of 8,720 earthquakes and first‐arrival times from 3,110 active source data (explosions and airguns) to construct a 3‐D seismic velocity model across California. Their model consists of eight layers (depths of 1, 4, 8, 14, 20, 27, 35, and 45 km) with a grid spacing of 10 km. We use the layer at a depth of 1 km, which is the shallowest layer available to constrain the bulk modulus for aquifer sediments that dominate the upper kilometer of the crust. The authors do not include a *V*_*S*_ model for the upper kilometer of the crust in the San Joaquin Valley, so we use a constant *V*_*P*_/*V*_*S*_ ratio of 1.73, which is given by the authors as a starting input into their final *S* wave velocity model. According to Hwang and Mooney (1986), who used seismic tomography to construct a seismic velocity model for the Central Valley, and as discussed by Lin et al. (2010), this ratio likely produces a *V*_*S*_ model that is too high for the San Joaquin Valley study area.

A multitude of studies besides the two mentioned above have examined *V*_*P*_, *V*_*S*_, and *V*_*P*_/*V*_*S*_ ratios both in situ and in laboratory experiments to try to derive analytical relationships for seismic velocities traveling through various mediums (e.g., Castagna et al., 1985; Lee et al., 1996; Uyanık, 2011). These studies have shown that *V*_*P*_ and *V*_*S*_ vary with porosity, saturation, confining pressure, and clay content. Shallow, unconsolidated, saturated, sedimentary units, and those with higher clay content display lower seismic velocities than dry, consolidated rock. Thus, the velocities derived from Lin et al. (2010) are likely overestimated in the Central Valley and may provide an upper‐bound estimate for the bulk modulus (which is discussed in section 4.3). If we consider that deformation is taking place primarily in the fine‐grained layers, then we can use an analytical relationship developed by Eberhart‐Phillips (1989) to estimate *V*_*P*_ and *V*_*S*_ in the Valley (km/s):

(3) $$V_{P}=5.77-6.94\varphi -1.73\sqrt{C}+0.446\left(P_{e}-1.0e^{-16.7P_{e}}\right)$$

(4) $$V_{S}=3.70-4.94\varphi -1.57\sqrt{C}+0.361\left(P_{e}-1.0e^{-16.7P_{e}}\right)$$

where *P*_*e*_ is effective pressure in kbars, *φ* is porosity, and *C* is clay volume fraction. Clay content varies spatially and with depth. However, in much of the San Joaquin Valley, it makes up more than 50% (Bertoldi et al., 1991; Faunt, 2009). Porosity values in the San Joaquin Valley range from ~0.34 to 0.43 (based on values compiled by Bertoldi et al., 1991). A back‐of‐the‐hand calculation using a porosity of 0.4, *C* = 0.5, and *P*_*e*_ = *ρgh* ≈ 1700 kg/m^3^ * 9.8 m/s^2^ * 1000 m ≈ 16.7 MPa ≈ 0.167 kbars, gives us *V*_*P*_ = 1.8 km/s, and *V*_*s*_ = 0.65 km/s, slightly lower than those found from Lin et al. (2010). Both the velocities from Lin et al. (2010) and those calculated using the Eberhart‐Phillips equations will be used to estimate the bulk modulus.

## 3 Methods: Volumetric Strain Rate Modeling

Volumetric strain at depth derived from surface displacements has been accomplished using leveling surveys (e.g., Vasco et al., 1988) and GPS (e.g., Mossop & Segall, 1999) as well as InSAR (e.g., Shirzaei et al., 2016; Vasco et al., 2002). Here, we follow the method of Mossop and Segall (1999) and solve for volumetric strain rate in an isotropic elastic half‐space by inverting vertical velocity measured at pixels displaying evidence of subsidence in the San Joaquin Valley on a three‐dimensional grid. We first down‐sample the dataset to reduce the number of InSAR pixels. To do this, we use a gradient‐based approach similar to the quadtree algorithm (Jonsson et al., 2002), adapted for irregularly sampled data points (Khoshmanesh et al., 2015). Following down‐sampling, we are left with 3,650 pixels with a spatial resolution of ~1.25 km × 2.5 km, representing the subsidence across the San Joaquin Valley.

The model grid consists of *m* = 8,250 triangular prism volume elements with sides of length ~6 km in the *x*‐direction, ~8 km in the *y*‐direction, and 200 m in the *z*‐direction from the surface to a depth of 1,000 m. The boundary constraints are set such that the strain rate goes to nearly zero at the edge of the model, including the last 800–1,000‐m depth volume elements. This depth range is chosen based on records of the deepest drilled wells and the aquifer sediment thickness. We solve for volumetric strain rate, *ε* = [*ε*_1_, …, *ε*_*m*_ ]^*T*^, at the center of each triangular prism {*X*_*i*_, *Y*_*i*_, *Z*_*i*_}, *i* = 1,2, …, *m*. The volumetric strain rate is assumed to be constant within each volume element (Mossop & Segall, 1999). Given surface displacements, *L* = [*L*_1_, *L*_2_, …, *L*_*n*_]^*T*^, we solve the following system of equations:

(5) $$\begin{array}{l} L=G\varepsilon +r\\ W={S_{0}}^{2}{C_{\mathrm{LL}}}^{-1} \end{array}$$

where *G* = [*G*_1_…*G*_*m*_] are the elastic Green's functions (Okada, 1992) scaled by the thickness of the prism. The observation residual is given by *r* = [*r*_1_… *r*_*n*_]^*T*^. *W* is the observation weight matrix, $C_{\mathrm{LL}}^{-1}$ is a diagonal matrix of the variance of VLM velocity, and $S_{0}^{2}$ is the primary variance factor, assumed to be 1 (Mikhail & Ackermann, 1982). The variance‐covariance matrix, *Q*, of the volume strain is

(6) $$Q=S_{0}^{2}{\left(G^{T}\mathrm{WG}\right)}^{-1}$$

The second derivative of *ε* is also minimized to avoid unrealistic volume strain (Harris & Segall, 1987).

To find a balance between the roughness of the modeled strain and its fit to the data, we also introduce a smoothing factor, *λ*. The optimal value for the smoothing factor is found using a trade‐off curve, in which we test a range of *λ* values and choose a value that provides a balance between the L2‐norms of model misfit and roughness. Given that the model is shallow, and observations provide good coverage, the model is well‐constrained and different values of *λ* have little effect on the model roughness and data fit.

**Figure 3:**
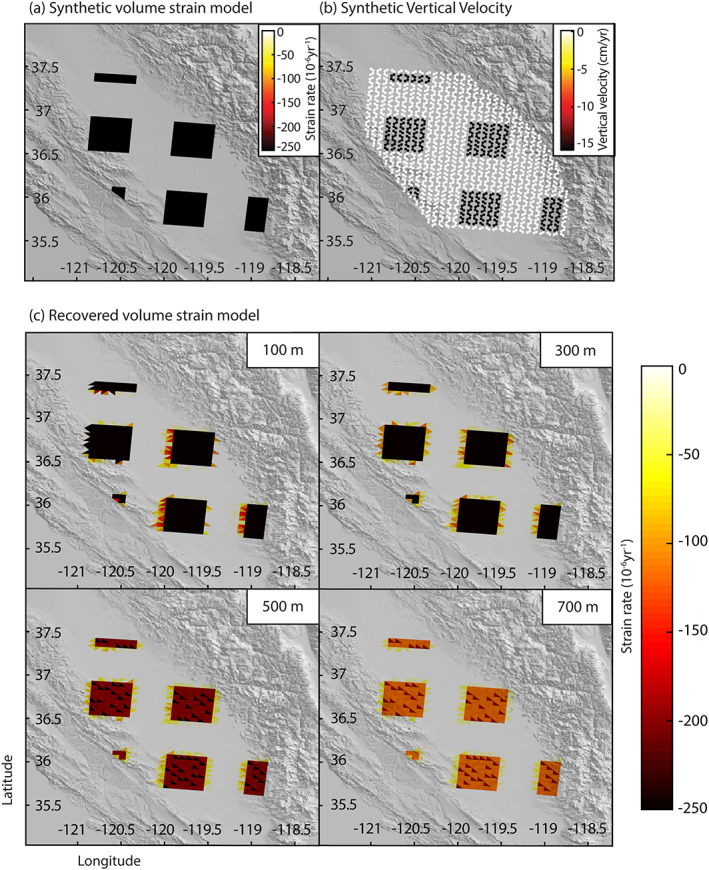
(a) Synthetic volume strain rate model for layer depths 0–800 m. Model layer 800–1,000 m is constrained to nearly zero. (b) Synthetic vertical velocity. (c) Resolved strain rate model for volumetric strain model at 100, 300, 500, and 700‐m depths. The model resolves the shallowest layers well, but resolution degrades with depth.

We use a synthetic test to analyze the model resolution obtained through our inversion method (Mossop & Segall, 1999). We create a synthetic strain rate model in a checkerboard pattern, as shown in Figure 3, with strain rates of −250 × 10^−6^ from the surface to 800 m depth. This model is used to generate synthetic observations of surface displacement. Using the inversion scheme described above, the synthetic observations from this volume strain rate model are used to solve for volumetric strain rate at depth. Figure 3 shows the recovered volume strain rate model at depths of 100, 300, 500, and 700 m. The inversion is able to resolve the volumetric strain well at shallow depth. However, because we have no way to constrain volumetric strain to a certain depth and because the inversion procedure systematically puts the strain closer to the surface, the modeled strain rate underestimates the magnitude, particularly at depth. This means the volumetric strain rate model is a lower bound on the actual strain rate caused by groundwater withdrawal, consistent with that found by Mossop and Segall (1999) for the Geysers geothermal field.

## 4 Results and Discussions

### 4.1 Volumetric Strain

**Figure 4:**
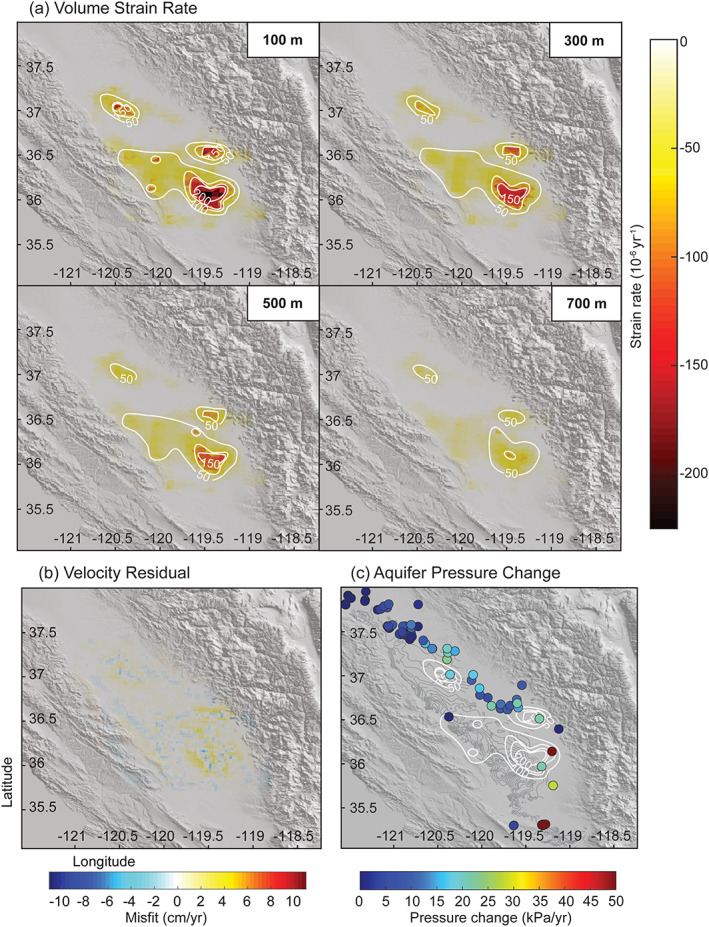
(a) Volume strain rate at 100, 300, 500, and 700‐m depths between 24 December 2006 to 1 January 2010. Contours are 50 micro‐strain per year. (b) Velocity residuals. Residuals are below ±5.1 cm/yr. (c) Aquifer pressure change rate in kPa/yr between 24 December 2006 to 1 January 2010 of wells either below or outside of the top of the Corcoran Clay overlain by volume strain rate contours at 100‐meter depth shown in (a) in white. Light gray contours are of the Corcoran Clay, same as Figure 2a.

Figure 4a shows volumetric strain rate at depths of 100, 300, 500, and 700 m depth derived from VLM observations between 24 December 2006 to 1 January 2010. Volumetric strain rate reaches a maximum of ~−150 × 10^−6^ yr^−1^ to −200 × 10^−6^ yr^−1^ at a depth of 0–200 m within three different subsiding bowls. An absolute maximum of −232 × 10^−6^ yr^−1^ occurs within the subsiding bowl near latitude, longitude [36.1, −119.5] which is located in Tulare and Kings County. The magnitude of strain decreases with depth in our model, but the pattern remains the same. We can conclude that strain is highly localized spatially and with depth within ~130 × 75 × 0.5 km^3^ volume in the San Joaquin Valley with two smaller bowls of subsidence to the north and northwest in Fresno and Merced Counties, respectively. These subsiding bowls experience a volume strain rate of up to ~−150 × 10^−6^ yr^−1^. Additionally, from our resolution test, we know that our model underestimates the strain rate at depth. Therefore, we can think of the strain rate estimates as a lower bound on the strain rate past ~400 m depth, particularly to the west where aquifer sediments are thicker and the Corcoran Clay forces pumping from deeper wells. Velocity residuals, *r*, are found by solving *r* = *L* − *Gε*. Residuals are small across the study area, with the largest found on the periphery of the subsiding zone (Figure 4b). This may be due to horizontal displacements caused by differential subsidence that are not accounted for in the model.

Fine‐grained layers vary laterally in the San Joaquin Valley, which may cause inhomogeneity of aquifer properties both horizontally and vertically. This, in turn, may cause differential subsidence rates, which could explain the pattern of the volumetric strain rate present in our model. Alternatively, this can show us where the greatest compaction is occurring due to the largest withdrawal rates, which may not necessarily correspond to locations of monitoring wells. Our assessment of pumping well distribution (CDWR, 2019a, 2019b) shows fewer wells in the region of greatest volume strain (supporting information Figure S1). This could reveal either that wells that are present are rapidly extracting groundwater during this time or that there is a lack of information regarding wells in these locations. Jeanne et al. (2019) showed that subsiding regions during both the 2007–2010 and 2012–2015 droughts also had high agricultural water demand. Additionally, many of the pumping wells analyzed by Jeanne et al. (2019) had depths between ~150 and 300 m, while most of our observation wells have depths shallower than 150 m (Figures 2 and S2). Figure 4c reveals that most of the deeper groundwater level observations are located to the north of the subsiding bowls, and there are very few groundwater level observations within these zones. Based on the assessment of Jeanne et al. (2019) as well as our own, we suspect that the observation wells with available time series during this period likely did not capture the complete groundwater level change in the rapidly subsiding bowls. Thus, our volume strain rate model likely provides a more reliable picture of contraction due to pumping at depth than can be gleaned from the groundwater observation well data alone.

### 4.2 Mapping Extensional Fissures

Fissures form when tensional stress from subsidence overcomes the cohesive strength of rock, soil, or sediment. We use our volumetric strain model to evaluate the risk of pumping‐induced surface extensional fissures. Fissures often begin as hairline fractures at depth. As tension increases or erosion of the crack ensues, they may develop significant cavities and collapse. Fissure development in a specific location may also be encouraged or discouraged by sediment type (Holzer & Bluntzer, 1984), strain accumulation along pre‐existing planes of weakness (Burbey, 2002; Sheng et al., 2003), or hydraulic seepage forces (Lofgren, 1971). Inside of subsiding zones, the rock and soil experience compression. However, on the periphery of subsiding regions, bending creates tensional stress as the material is pulled towards the center of the subsiding zone creating vertical, elongate surface cracks (Holzer & Bluntzer, 1984; Holzer & Pampeyan, 1981).

We use the ratio of minor principal stress to sediment tensile strength to evaluate the risk of fissure initiation or propagation (in the case of preexisting cracks). This ratio, *R*, is given by Sheng et al. (2003):

(7) $$R=\frac{\sigma_{3}}{\tau_{s}}$$

where *σ*_3_ is the minor principal stress and *τ*_*s*_ is the tensile strength. We adopt the convention that *σ*_3_ is negative for tension and positive for compression. The tensile strength of soil can vary depending on the soil particle sizes, water content, and compressive stress. As compression increases, percent clay increases, and water content is close to a critical value (~11.5%), the tensile strength increases (Tang et al., 2015). Here, we are interested in the tensile strength of the unsaturated or semi‐saturated vadose zone, which lies above the aquifer and is relatively dry and shallow. Tensile strength tests done on soil masses by Conwell (1965) as cited by Sheng et al. (2003) and Hernandez‐Marin and Burbey (2010) and Tang et al. (2015) show values ranging from ~1 × 10^4^ to 9 × 10^4^ Pa. We select a value of *τ*_*s*_ = 8 × 10^4^ Pa based on the degree of compaction and higher clay content of subsoil in parts of the Central Valley (USDA/NRCS, 2006) as well as the presence of cemented duripan or hardpan soil layers in parts of the San Joaquin Valley with higher tensile strength (USDA/NRCS, 1988, 2003). Results using a range of tensile strengths are given in the supporting information.

Using the cumulative volumetric strain over the 3‐year drought period, we calculate the 3‐D strain tensor near the surface at a depth of 1 meter in the unsaturated vadose zone to evaluate the risk of tensile fissuring (Comninou & Dundurs, 1975). We then convert this to stress using a linear transformation of Hooke's law. To obtain a value for *σ*_3_, we solve for the eigenvalues of a 2‐D stress tensor, [*σ*_*xx*_ *σ*_*xy*_ *σ*_*yx*_ *σ*_*yy*_ ], providing the maximum, *σ*_1_, and minimum, *σ*_3_, horizontal stresses.

**Figure 5:**
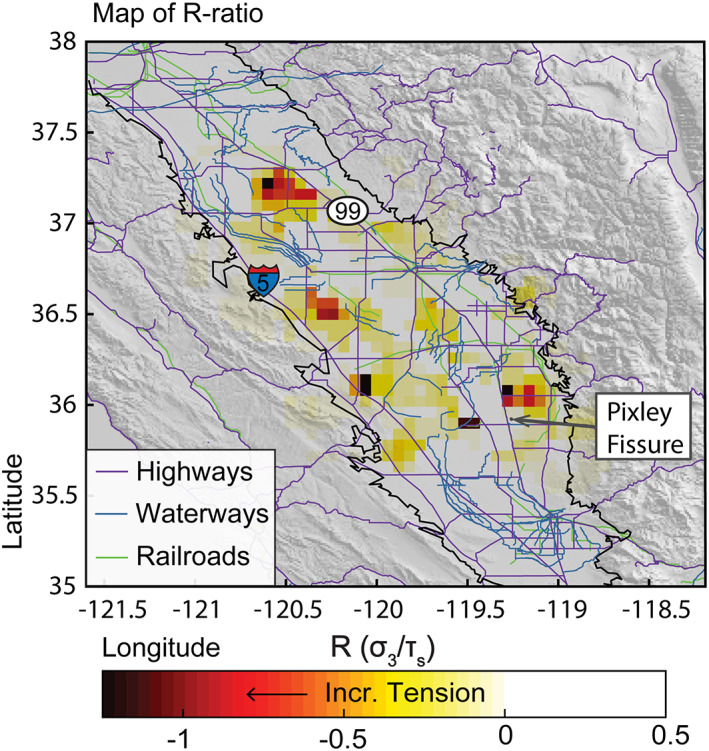
Map of predicted *R*‐values, which is the ratio between minimum principal stress and soil strength. Negative values show increasing tension. Darker red colors show a higher risk of fissure initiation. Plot overlain with highways, railroads, and local canals and aqueducts (waterways) in California. Interstate 5 and California State Route 99 indicated by 5 and 99, respectively. The San Joaquin Valley is outlined in black. The Pixley fissure, identified in 1969, is indicated in gray.

A map of predicted *R* values showing the ratio of minor principal stress to soil tensile strength is shown in Figure 5. The largest tensile stresses are found on the periphery of the subsiding zone and are especially high near latitude, longitude [36, −119.2] in Tulare County and latitude, longitude [37, −120.5] in Merced County. In general, compressive stresses dominate within the different subsiding bowls (shown by a lack of color in the center of Figure 5). The pattern is irregular due to the shapes and close spacing of the subsiding bowls. The highest tension occurs near the edge of the basin where there is already increased risk of fissure development because aquifer system sediments thin towards the edges, creating differential aquifer thickness not reflected in our model, but susceptible to additional differential compaction (Jachens & Holzer, 1982). This is likely a more significant threat in Tulare County than regions on the western edge of the Valley because sediments are thinnest on the eastern margin of the basin.

There are no widely available field maps of fissure locations; therefore, we cannot easily validate our results. The Pixley fissure location (Figure 5) lies just outside of a large, negative *R*‐ratio zone. At the time of its discovery in 1969, the Pixley fissure was located on the eastern margin of a small subsidence bowl near Pixley, CA, that has since recovered (Rucker et al., 2019; Lofgren & Klausing, 1969). The lack of evidence of surface fissures may be due to high levels of soil and subsoil alteration from agriculture or lack of reporting of surface fissures on private farmland. Fissures that begin as hairline fractures at depth may be continuously infilled or covered at the surface with soil and sediment from deep tillage or land leveling practices, which are common in the San Joaquin Valley (USDA/NRCS, 2006, 2003). In some places outside of the Central Valley where fissures have been reported, the location and orientation of tensile fissures are often controlled by basement structural inhomogeneities or rapid changes in aquifer compressibility due to thick aquitard lenses. For example, this is the case for fissures in central Phoenix, Arizona, USA, which dominantly form between bedrock highs and rapidly subsiding basins (e.g., Borchers et al., 2014; Jachens & Holzer, 1982). The San Joaquin Valley sediment basin is thick and contains few significant structural inhomogeneities. Thus, it would not likely play a role in possible fissure development. However, differential aquifer compaction due to variable Corcoran Clay or other clay layer thickness could play a role. Detailed mapping of the locations of clay lenses and their thicknesses, as well as aquifer pressure change, may help determine the risk of differential compaction in the Valley. Moreover, in the vicinity of the high‐volume wells, shear failure at depth can act as a hydrologic barrier, concentrating tensile stress on one side of the fault, which results in upward migrating tensile cracks (Sheng & Helm, 1998). Such cracks take a long time to reach the surface, which may explain why surficial fissures are scarce in San Joaquin Valley, despite recent excess pumping.

In addition to fissuring, areas of increased tension are at risk for tension cracking on canals, roads, railways, pipelines, and other infrastructure. According to our estimation (Figure 5), several highways, including Interstate 5 and California State Route 99, and some stretches of canals and aqueducts are at risk for tensile cracking. Lack of documentation on tension cracking and fissure initiation and propagation in the San Joaquin Valley makes this an important, yet under‐examined problem. With the present population rise, infrastructure and canal development will likely increase. This expected infrastructure development coupled with inevitable recurrent drought and increases in groundwater use will make hazard prediction and assessment of fissure development increasingly essential.

### 4.3 Constraining the Bulk Modulus

An important poroelastic parameter is the bulk modulus, which is defined as the reciprocal of the rock compressibility. Ramey et al. (1974) shows that within the temperature range of 24°C and 205°C, the rock bulk modulus (compressibility) increases (decreases) when pressure increases from 0.1 to 55 MPa. Often seismic velocity profiles are used to estimate the bulk modulus (Mavko et al., 2009). Such estimates correspond with a high‐frequency deformation rate of 10–100 Hz, thus are called the dynamic bulk modulus (*K*_*dyn*_). Deformation in this case occurs in an “undrained” state where deformation is too fast for fluid to escape, resulting in an increase or decrease in pore pressure. The dynamic bulk modulus is estimated using an equation involving *P* wave velocities (*ν*_*p*_), *S* wave velocities (*ν*_*s*_), and sediment density (*ρ*)

(8) $$K_{\mathrm{dyn}}=\rho \left(\nu_{p}^{2}-\frac{3}{4}\nu_{s}^{2}\right)$$

The corresponding values of the dynamic bulk modulus for the San Joaquin Valley using seismic velocity profiles provided by Lin et al. (2010) range from 10.9 to 51.9 GPa. According to Lin et al. (2010) and as discussed in section 2.3, seismic velocity values are slightly overestimated near the surface; thus, our dynamic bulk modulus estimate may also be slightly exaggerated. We also calculate the dynamic bulk modulus for a value of *V*_*p*_ = 1.8 km/s and *V*_*S*_ = 0.65 km/s as estimated using the Eberhart‐Phillips (1989) equations (Equations 3 and 4), which are likely more appropriate in sedimentary basins. This results in a slightly lower estimate for the dynamic bulk modulus, *K*_*dyn*_ = 5.6 GPa (Table 1).

Assuming the deformation associated with the process of fluid extraction is of a low‐frequency, one can consider a “drained” condition where the deformation process is slow enough that fluid can flow into and out of the pore space, resulting in constant pore pressure. Thus, the effective drained Bulk modulus (*K*_*eff*_) might be more representative of a slow deformation process, like subsidence, with a long‐term equilibrium pore pressure and is defined by Wang (2000):

(9) $$K_{\mathrm{eff}}=K_{\mathrm{dyn}}*\left(1-\alpha \beta \right)$$

where *α* is the Biot‐Willis coefficient and *β* is the Skempton coefficient. The Biot‐Willis coefficient represents the proportion of fluid pressure that can counteract the confining stress and can range from 0 to 1 based on how confined (1) or unconfined (0) the aquifer is. We set *α* = 0.85 because the San Joaquin Valley aquifer system is semi‐confined (east side, where the Corcoran Clay is absent) to confined (west side, where the Corcoran Clay is present). Using this value, we can solve for *β* using the drained (*ϑ*) and undrained (*ϑ*_*u*_) Poisson ratios:

(10) $$\alpha \beta =\frac{3\left(\vartheta_{u}-\vartheta \right)}{\left(1-2\vartheta \right)\left(1+\vartheta_{u}\right)}$$

We set *ϑ*_*u*_ = 0.31 based on values given in Table C1 in Wang (2000) and *ϑ* is solved for using

(11) $$\vartheta =\frac{1}{2}*\left(1-\frac{1}{{\left(\frac{\nu_{p}}{\nu_{s}}\right)}^{2}-1}\right)$$

**Figure 6:**
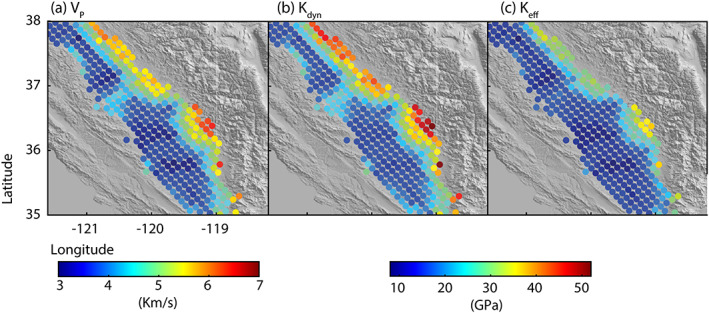
Estimated bulk moduli in the San Joaquin Valley. (a) *P* wave seismic velocity from Lin et al. (2010) at 1 km depth. (b) Dynamic bulk modulus based on seismic velocities in (a) for high‐frequency deformation. (c) Effective drained bulk modulus based on Equation 9 for a slow deformation process, like subsidence, with a long‐term equilibrium pore pressure. Bulk moduli are overall smaller on the western side of the Valley, where valley‐fill sediments are thickest. Range of the dynamic bulk modulus and the effective drained bulk modulus are listed in Table 1.

Values for the effective bulk modulus are given in Figure 6c and range from 7.9 to 37.7 GPa when using the Lin et al. (2010) seismic velocities and 4.1 GPa when using the Eberhart‐Phillips (1989) equations (Equations 3 and 4).

We can also solve for the quasi‐static bulk modulus (*K*), given our strain rate and pore pressure rate (Figures 4a and 2b). The relation between volumetric strain (*ε*_*v*_), pore pressure (*σ*_*p*_), and *K* is (Mossop & Segall, 1999; Nur & Byerlee, 1971)

(12) $$\frac{1}{K}=\frac{1}{K_{0}}+\frac{{\Delta \varepsilon }_{v}}{{\Delta \sigma }_{p}}$$

where *K*_0_ is the mineral bulk modulus, which is the bulk modulus of the grains only, set equal to 42 GPa based on Table C1 in Wang (2000) for sandstone and mudstone. In this case, pore pressure is “quasi‐static.” This means that changes in the pore pressure, due to groundwater pumping, induce stress on the sediment matrix and drives local contraction of the bulk sediment volume, but also accounts for flow into and out of the pore space to re‐equilibrate the pressure. As was discussed earlier, the strain rate model tends to underestimate the volumetric strain rate at depth. Thus, we are able to place a lower bound on the value for the quasi‐static bulk modulus, *K*. We consider that *ε*_*v*_ > 2.3 × 10^−4^ yr^−1^, the maximum volumetric strain rate at a depth of 100 m. Δ*σ*_*p*_ is computed using the average pressure change rate of two wells (023S025E06C002M and 021S026E06A001M) that fall within the largest zone of contraction and are either beneath or outside of the clay layer, resulting in Δ*σ*_*p*_ = 44.7 kPa/yr. This produces an estimate for the quasi‐static bulk modulus of *K* = 0.19 GPa. This bulk‐modulus estimate is smaller than the dynamic bulk modulus calculated using seismic velocity and our estimated *K*_*eff*_, which ranges from 4.1 to 37.7 GPa (Table 1).

**Table 1:** Summary of Estimated Bulk Moduli

	Conditions under which the moduli apply		Minimum value (GPa)	Maximum value (GPa)
	Speed of deformation	Pore pressure		
*K*_*dyn*_	Rapid deformation	Undrained	5.6	51.9
*K*_*eff*_	Slow deformation	Drained	4.1	37.7
*K*	Slow deformation	Quasi‐static	0.19	—

*Note*. Minimum values for *K*_*dyn*_ and *K*_*eff*_ are derived from the Eberhart‐Phillips (1989) equations (Equations 3 and 4). The quasi‐static bulk modulus is most representative of the pore‐pressure and deformation conditions of aquifer compaction.

## 5 Conclusions

Subsidence continues in the San Joaquin Valley due to the compaction of aquifer fine‐grained material. During the 2007–2010 drought, compaction caused a volume strain rate of up to −232 microstrain/yr in one of the regions hardest hit by groundwater extraction and subsidence, the San Joaquin Valley. This value is likely a lower bound estimate, and volume strain rates may, in reality, be greater, particularly at depth. When comparing our pressure change estimates derived from groundwater level observations with our volume strain rate model, we find that the largest pressure changes occur on the edges of the largest subsiding zones, but we do not have groundwater level observations within these zones of greatest contraction. This indicates that the highest groundwater extraction rates are not recorded during the studied drought period. Thus, aquifer pressure change might, in reality, be larger than is estimated in this study.

Contraction within subsidence bowls in the San Joaquin Valley creates tensile stress that, in some cases, may overcome sediment or soil strength, leading to surface fissures and tensile cracking. The periphery of the regions experiencing the most dramatic subsidence is at the highest risk for fissure initiation, particularly at approximate latitude, longitude [37, −120.5] and [36, −119.2] in Merced and Tulare Counties, respectively, where *R*‐ratios are more negative than −1. Additionally, a number of segments of highways and waterways are at risk of tensile cracking. Because these form outside of the regions of greatest subsidence, we note that hazards associated with groundwater overdraft are further reaching than the local subsiding bowls alone. Ongoing, and likely future groundwater overdraft will continue to create hazards related to aquifer compaction like fissuring and tension cracking. Determining those places most at risk is important for infrastructure planning and hazard assessment.

Aquifer compaction occurs over much longer time scales than deformation caused by the propagation of seismic waves passing relatively fast through the medium. Thus, the poroelastic variable used to describe a slow deformation process, like subsidence, is one to two orders of magnitude smaller than that for high‐frequency deformations determined using seismic velocity data. The bulk modulus calculated here is useful in the case of quasi‐static pore pressure, where aquifer pressure change induces local volume strain, as is the case for groundwater withdrawal from the aquifer in the San Joaquin Valley. Using our volumetric strain rate and pore pressure calculations, we calculate a lower bound estimate for the quasi‐static bulk modulus of 0.19 GPa.

## Supporting information

Supporting Information S1

## Data Availability

V. L. M. data are obtained from Ojha et al. (2018). All groundwater observation data are obtained from the California Department of Water Resources (https://data.ca.gov/dataset/) and the U.S. Geological Survey (https://waterdata.usgs.gov/nwis/gw). Contours of depth of the Corcoran Clay obtained from the USGS (https://ca.water.usgs.gov/projects/central-valley/central-valley-spatial-database.html). Seismic velocity data are obtained from Lin et al. (2010).
