# Supplementary material for: Subsidence‐Derived Volumetric Strain Models for Mapping Extensional Fissures and Constraining Rock Mechanical Properties in the San Joaquin Valley, California
Source: J Geophys Res Solid Earth. 2020 Sep 11;125(9):e2020JB019980. doi: 10.1029/2020JB019980 (PMC7539920; doi:10.1029/2020JB019980)
Supplement: Supplementary file 1 — Supporting Information S1 [file JGRB-125-e2020JB019980-s001.docx]

*Journal of Geophysical Research: Solid Earth*

Supporting Information for

S**ubsidence-Derived Volumetric Strain Models for Mapping Extensional Fissures and Constraining Rock Mechanical Properties in the San Joaquin Valley, California**

G. Carlson,^1*^ M. Shirzaei^1,3^, C. Ojha^1,4^, S. Werth^1,2,3^

^1^ School of Earth and Space Exploration, Arizona State University, Tempe, AZ, USA

^2^ School of Geographical Sciences & Urban Planning, Arizona State University, Tempe, AZ, USA.

^3^Department of Geosciences, Virginia Tech, Blacksburg, VA, USA

^4^Positioning and Community Safety Division, Geoscience Australia, Canberra ACT 2601, Australia

**Contents of this file:**

Figures S1-S5

**Introduction**

Contained in this document is:

1. Locations of groundwater pumping wells (CDWR, 2019).
2. Boxplots of groundwater level rate change and observation well depths.
3. R-ratio plot overlain by volume strain rate contours in 4a.
4. R-ratio plots with soil tensile strengths of $1x{10}^{5} \mathrm{Pa}, 0.8x{10}^{5} \mathrm{Pa}, 0.5x{10}^{5} \mathrm{Pa},$

$0.2x{10}^{5} Pa, 1x{10}^{4} Pa$.

1. Effect of varying the Biot-willis coeffiecient, undrained poisson ratio, and Skempton coefficient on the drained, effective bulk modulus.


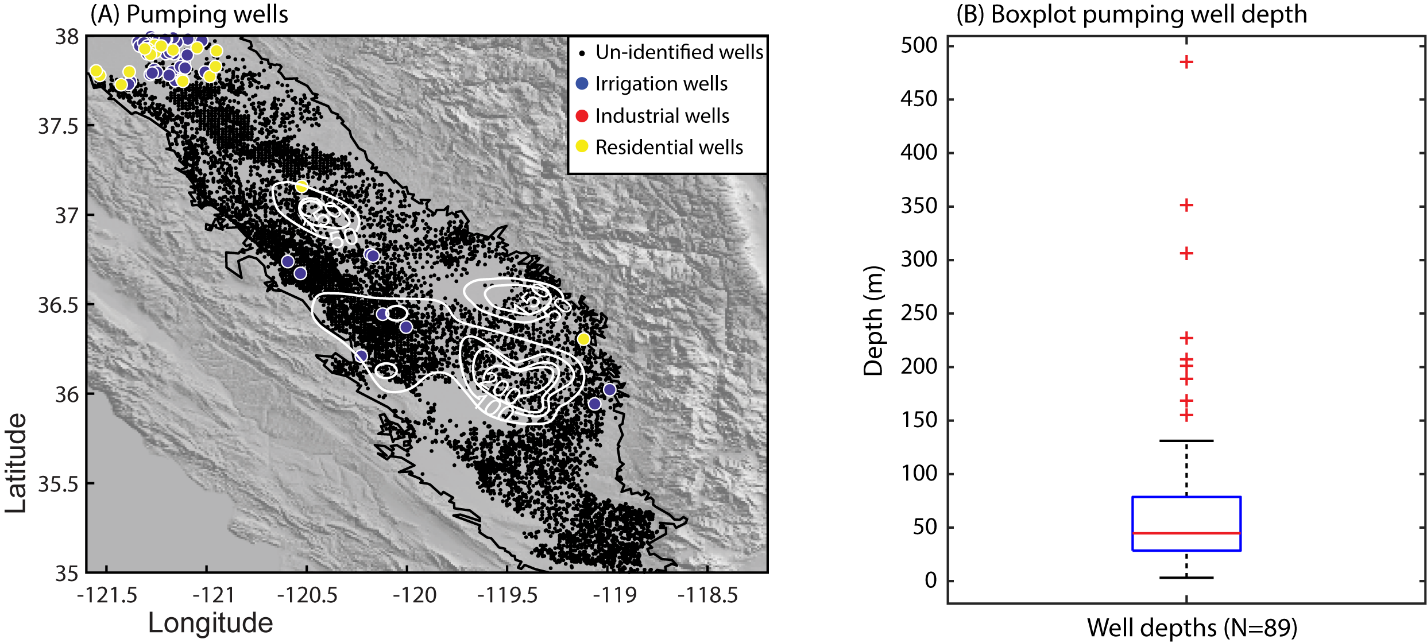


**Figure S1.** **(A)** Locations of groundwater pumping wells colored to use-type (Black-not specified, blue- irrigation wells, red- industrial wells, yellow-residential wells). White contours are of volume strain rate in micro-strain/yr at 100-meter depth same as Figure 4a. **(B)** Boxplot showing depth distribution of 89 pumping wells with listed depths. Median is shown by the red line. 25^th^ and 75^th^ percentiles are shown by the lower and upper limits of the blue box. Black solid lines show the end of the whiskers of the plot, which are 1.5x the interquartile length. Red crosses are outliers, which are outside of 1.5x the interquartile length.


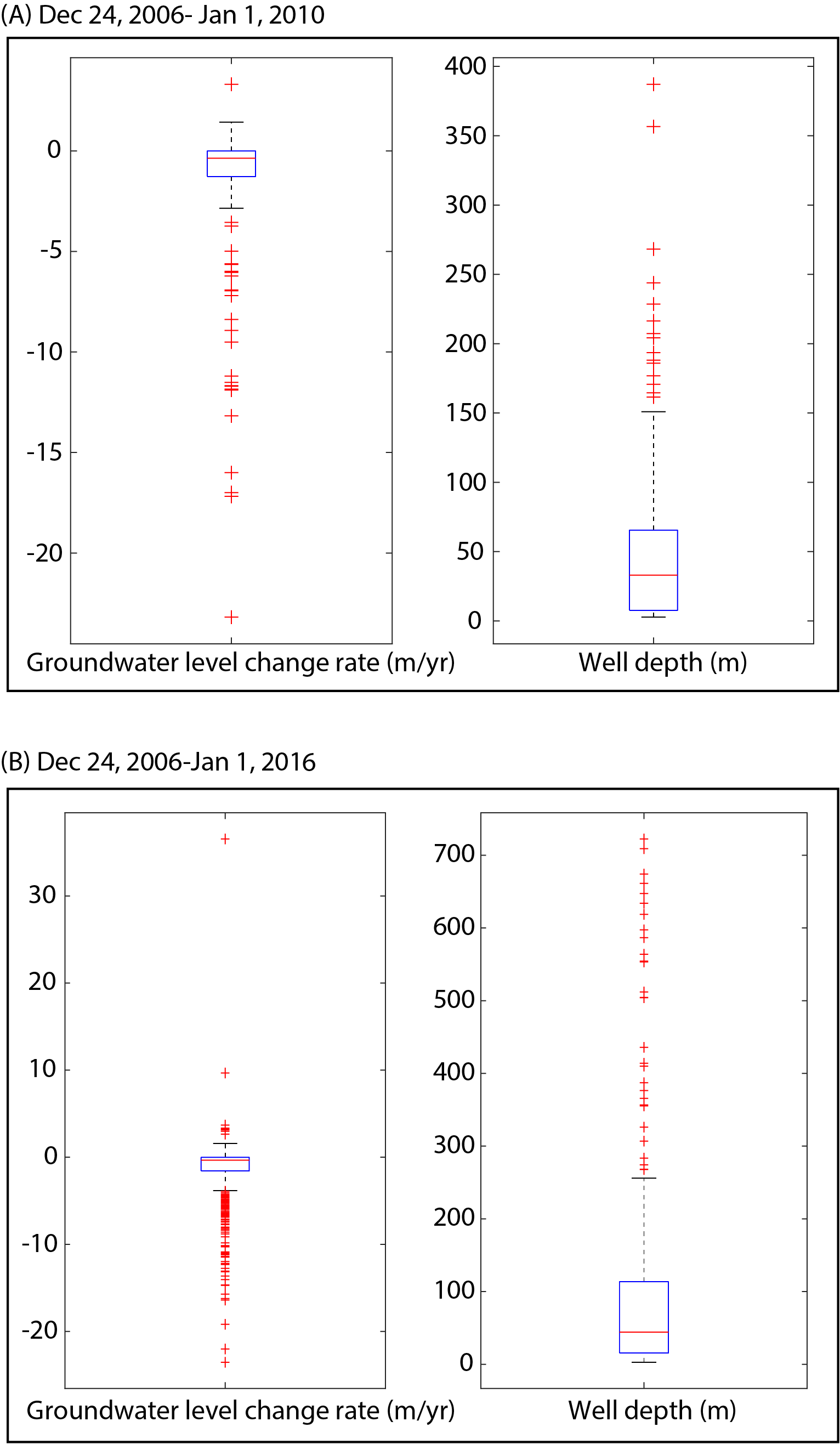


**Figure S2.** Boxplots of groundwater level rate change (panel 1) and well depth (panel 2) during **(A)** the first drought December 24, 2006-January 1, 2010 and **(B)** December 24, 2006-January 1, 2016, which is after and including the drought. Limits within each boxplot are the same as Figure S1B.


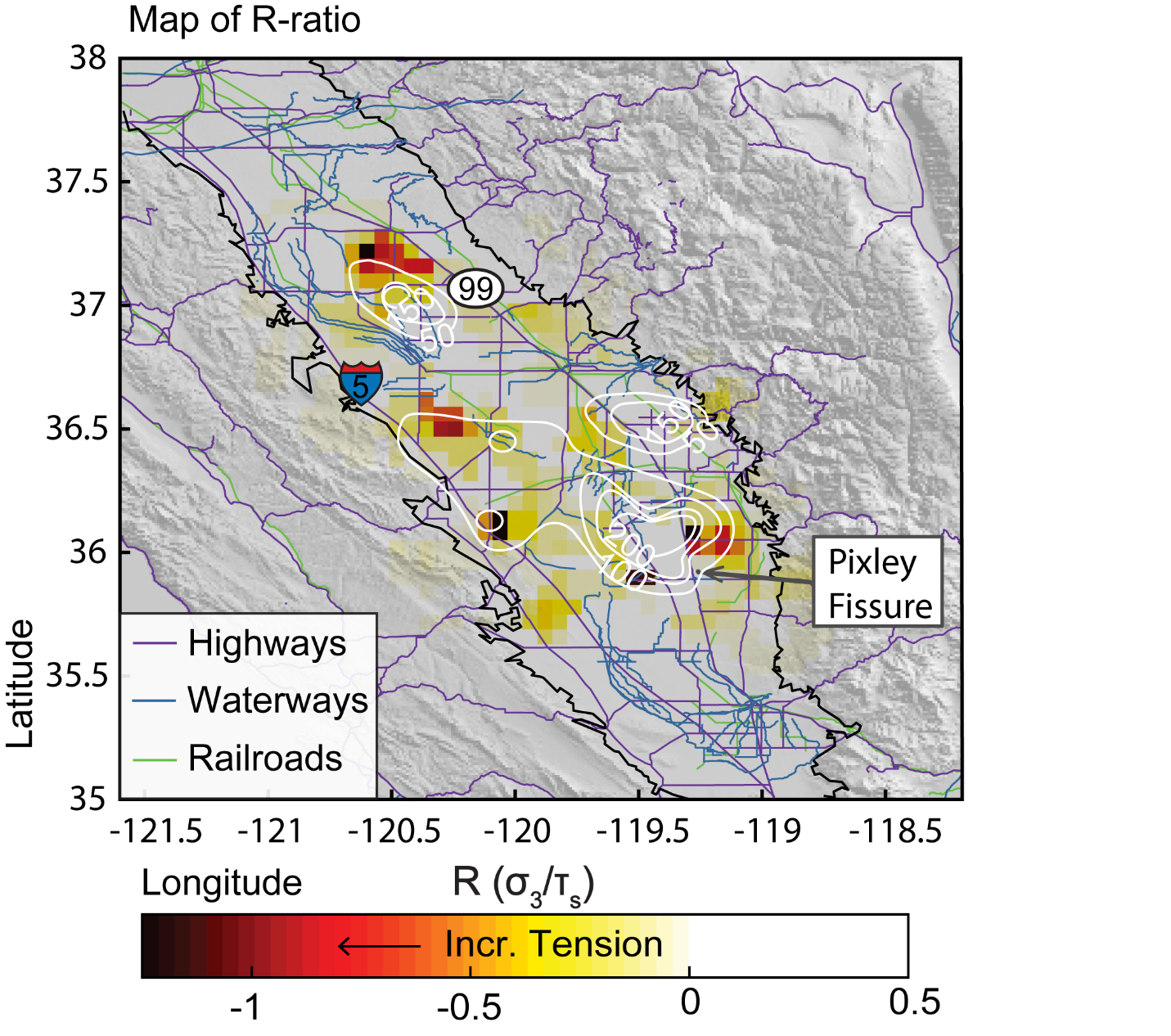


**Figure S3.** R-ratio plot same as Figure 5 overlain by white contours of volume strain rate in micro-strain/yr at 100-meter depth same as Figure 4a


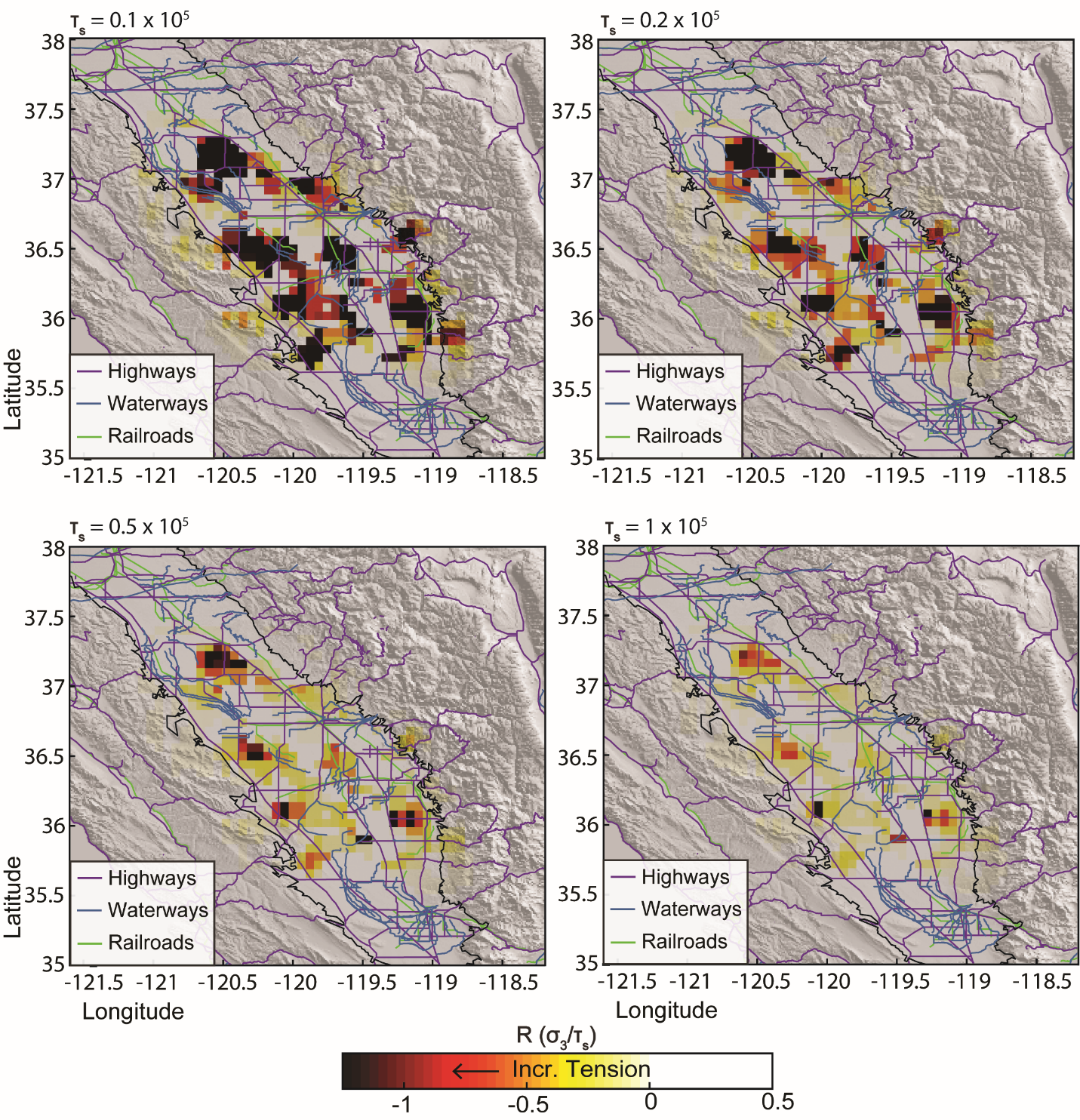


**Figure S4.** R-ratio plots with various tensile strengths calculated at 1m depth overlain by highways, waterways, and railroads. Tensile strength is in the header and given in Pa. Conwell (1965) measured tensile strength of soil at 0.1 x 10^5^ Pa (upper left), however this is likely a lower bound on the soil tensile strength due to the higher clay content and cemented hardpan soil layers that can be found in the San Joaquin Valley.


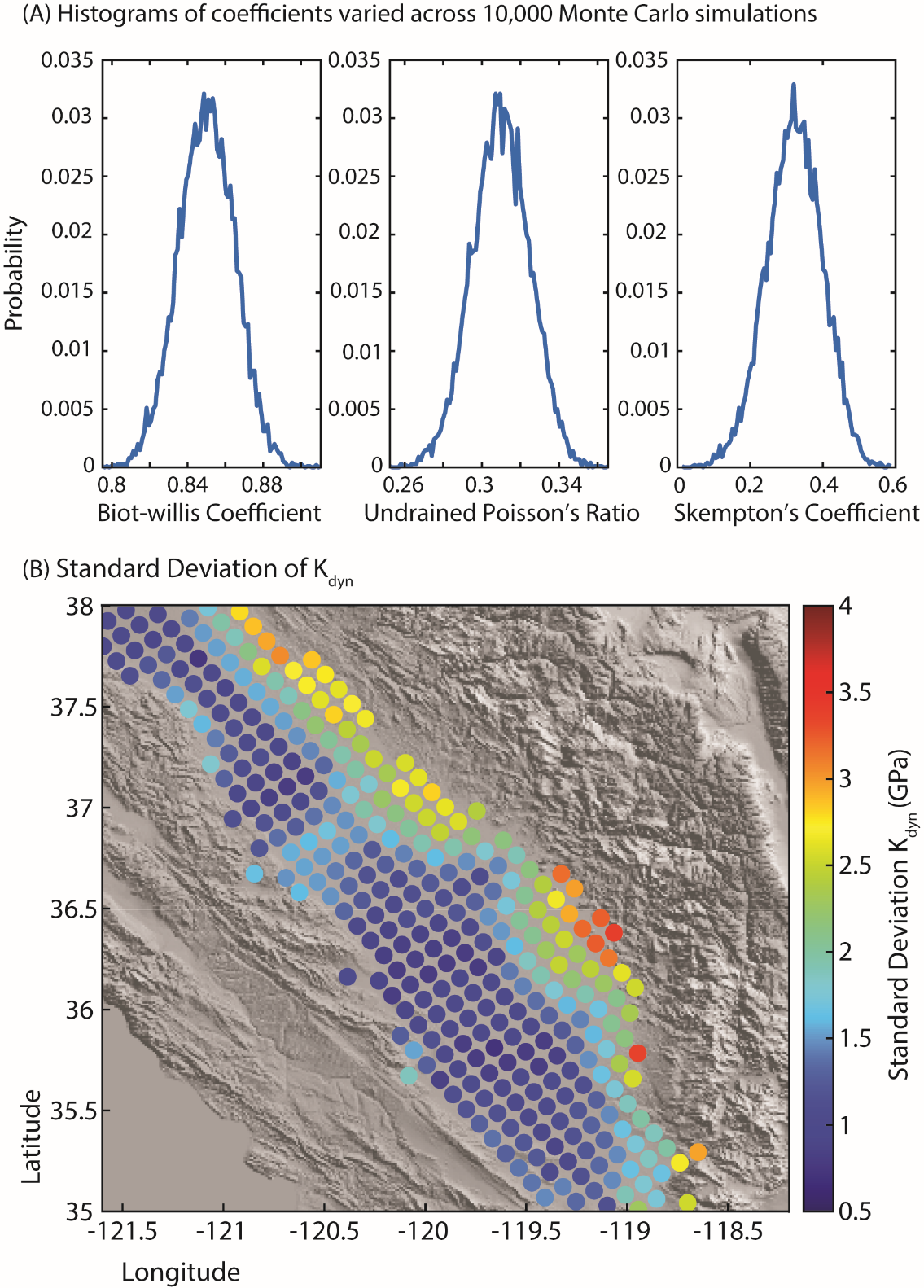


**Figure S5.** Monte-Carlo simulations showing the effect of varying the Biot-Willis coefficient, undrained Poisson’s ratio, and Skempton’s coefficient on the dynamic bulk modulus. The largest deviations occur on the eastern edge of the Valley where the Valley-fill sediments are thinner and the seismic velocities are higher (Figure 6).
